# Genome-resolved adaptation strategies of *Rhodobacterales* to changing conditions in the Chesapeake and Delaware Bays

**DOI:** 10.1128/aem.02357-24

**Published:** 2025-01-08

**Authors:** Mir Alvee Ahmed, Barbara J. Campbell

**Affiliations:** 1 Department of Biological Sciences, Clemson University 170362 , Clemson, South Carolina, USA; University of Delaware, Lewes, Delaware, USA

**Keywords:** *Rhodobacterales*, growth rate, metagenome-assembled genome, estuarine, genomospecies

## Abstract

The abundant and metabolically versatile aquatic bacterial order, *Rhodobacterales*, influences marine biogeochemical cycles. We assessed *Rhodobacterales* metagenome-assembled genome (MAG) abundance, estimated growth rates, and potential and expressed functions in the Chesapeake and Delaware Bays, two important US estuaries. Phylogenomics of draft and draft/closed *Rhodobacterales* genomes from this study and others placed 46 nearly complete MAGs from these bays into 11 genera, many were not well characterized. Their abundances varied between the bays and were influenced by temperature, salinity, and silicate and phosphate concentrations. *Rhodobacterales* genera possessed unique and shared genes for transporters, photoheterotrophy, complex carbon degradation, nitrogen, and sulfur metabolism reflecting their seasonal differences in abundance and activity. *Planktomarina* genomospecies were more ubiquitous than the more niche specialists, HIMB11, CPC320, LFER01, and MED-G52. Their estimated growth rates were correlated to various factors including phosphate and silicate concentrations, cell density, and light. Metatranscriptomic analysis of four abundant genomospecies commonly revealed that aerobic anoxygenic photoheterotrophy-associated transcripts were highly abundant at night. These *Rhodobacterales* also differentially expressed genes for CO oxidation and nutrient transport and use between different environmental conditions. Phosphate concentrations and light penetration in the Chesapeake Bay likely contributed to higher estimated growth rates of HIMB11 and LFER01, respectively, in summer where they maintained higher ribosome concentrations and prevented physiological gene expression constraints by downregulating transporter genes compared to the Delaware Bay. Our study highlights the spatial and temporal shifts in estuarine *Rhodobacterales* within and between these bays reflected through their abundance, unique metabolisms, estimated growth rates, and activity changes.

**IMPORTANCE:**

In the complex web of global biogeochemical nutrient cycling, the *Rhodobacterales* emerge as key players, exerting a profound influence through their abundance and dynamic activity. While previous studies have primarily investigated these organisms within marine ecosystems, this study delves into their roles within estuarine environments using a combination of metagenomic and metatranscriptomic analyses. We uncovered a range of *Rhodobacterales* genera, from generalists to specialists, each exhibiting distinct abundance patterns and gene expression profiles. This diversity equips them with the capacity to thrive amidst the varying environmental conditions encountered within dynamic estuarine habitats. Crucially, our findings illuminate the adaptable nature of estuarine *Rhodobacterales*, revealing their various energy production pathways and diverse resource management, especially during phytoplankton or algal blooms. Whether adopting a free-living or particle-attached existence, these organisms demonstrate remarkable flexibility in their metabolic strategies, underscoring their pivotal role in driving ecosystem dynamics within estuarine ecosystems.

## INTRODUCTION

The *Rhodobacterales* is one of the predominant and versatile orders found in both particle-attached (PA) and free-living (FL) fractions of aquatic environments, particularly in marine ecosystems. They also serve as important models for understanding marine microbial ecology (1–5). Previous studies, employing microscopy or culture-dependent approaches and 16S rRNA gene-based analyses, along with more recent ‘omics studies, have highlighted the abundance and diverse metabolic activities of *Rhodobacterales* in marine waters and sediments (1, 6–8). However, limited research exists on the temporal and spatial taxonomic and functional variations of *Rhodobacterales* in estuarine environments (9).

The remarkable metabolic versatility of *Rhodobacterales* allows them to thrive in various marine ecosystems, including temperate and polar oceans, coastal areas, and deep sea environments (1). They may constitute a significant proportion of bacterial communities, accounting for up to 20% and 5%, respectively, of communities in coastal ecosystems and open ocean surface waters and are mostly associated with algal blooms (10). A well-studied clade within the *Rhodobacterales*, *Roseobacter*, uses different energy production and carbon acquisition mechanisms, including carbon monoxide, sulfur transformation abilities, hydrogen sulfide oxidation, and hydrocarbon degradation (8, 11–13). Some strains utilize bacteriochlorophyll *a* to capture light energy, while others rarely possess xanthorhodopsin or proteorhodopsin for light-driven processes (1).

The Chesapeake and Delaware Bays, located in the Mid-Atlantic region of the USA, offer excellent opportunities to study the diversity of microbes in different ecological niches. The Chesapeake Bay, the largest estuary of the United States (14, 15), exhibits strong salinity gradients where it receives substantial freshwater inflow, sediment deposition, and particles (16, 17). The bay is highly stratified, especially in summer (18). Factors such as temperature and substrate availability are key controls on bacterial abundance and production in the bay (16, 19). Phytoplankton production varies seasonally due to light availability and is influenced by nitrogen inputs from the Susquehanna River (20). On the other hand, the Delaware Bay receives 51% of total freshwater input from the Delaware River (17, 21). Unlike the Chesapeake Bay, the Delaware Bay is generally well mixed, and primary production is limited despite high nutrient concentrations because of high turbidity and reduced light penetration (22, 23). In the summer, urban inputs cause higher nitrogen and phosphorus concentrations in the Delaware in contrast to the Chesapeake Bay, where ammonium and phosphate concentrations typically increase through bottomwater regeneration. The Chesapeake has overall lower nitrate concentrations than the Delaware Bay, and the latter supports larger spring blooms as evidenced by chlorophyll *a* concentrations (24, 25).

Microbial communities in these bays vary with site-specific characteristics, and more importantly with seasonal variations, and their composition changes mostly with salinity and temperature (26, 27). Higher diversity indices in the upper Chesapeake Bay than in the lower Bay were identified in one prior study (28). *Alphaproteobacteria*, *Betaproteobacteria*, *Gammaproteobacteria*, *Cyanobacteria*, *Actinobacteria*, *Planctomycetes*, and *Bacteroidetes* were common in different seasons. Within the *Alphaproteobacteria*, SAR11 was particularly abundant in warm waters, whereas *Roseobacter* thrived in cold waters (19). In the Delaware Bay, microbial communities also changed along the salinity gradient. In the freshwaters of the estuary, *Actinobacteria*, *Verrucomicrobia,* and *Betaproteobacteria* dominated, while high salinity coastal waters contained high abundances of SAR11, and lower abundances of *Rhodobacterales*, *Gammaproteobacteria* and *Bacteroidetes* (27).

We propose that differences in salinity, freshwater input, turbidity and light penetration, nutrient availability, and physico-chemical parameters between the Chesapeake and Delaware Bays play a significant role in shaping *Rhodobacterales* composition, growth, and function in these estuaries. The greater stratification and longer retention time in the Chesapeake compared to the Delaware estuary also may create conditions conducive to the development of a more estuary-specific microbial community. We explored how spatial and temporal environmental gradients shape the ecology of *Rhodobacterales* in estuarine systems and we found ubiquitous members like *Planktomarina*, and specialists like HIMB11, LFER01, or MED-G52 in samples from various salinity and seasons from two distinct bays.

## RESULTS

### *Rhodobacterales* metagenome-assembled genome

In total, 12 and 24 water samples were collected from the Chesapeake and Delaware Bays, respectively, for metagenome-assembled genome (MAG) construction. Delaware and Chesapeake Bay samples were collected during the spring, summer, and fall of 2014 or the spring and summer of 2015, respectively, from low (0.019–0.06 PSU), medium (15–22 PSU), and high (27–31 PSU) salinities (29, 30). For labeling, the first two letters of a sample name, like CPSpr15L08 or DESum29G08 indicated the bay (CP or DE for the Chesapeake and the Delaware Bay, respectively), followed by letters for the season of collection (Spr and Sum for Spring and Summer, respectively, and Fall), then numbers indicating salinity in PSU, and finally G08 or L08 to indicate larger (>0.8 µm) or smaller (<0.8 µm) size fractions. In addition, a D or N indicated samples collected during the day or at night, respectively. The MAG names were distinguished with an underscore after the metagenome label followed by associated bin numbers (e.g., DESpr20G08_bin_28).

**Fig 1:**
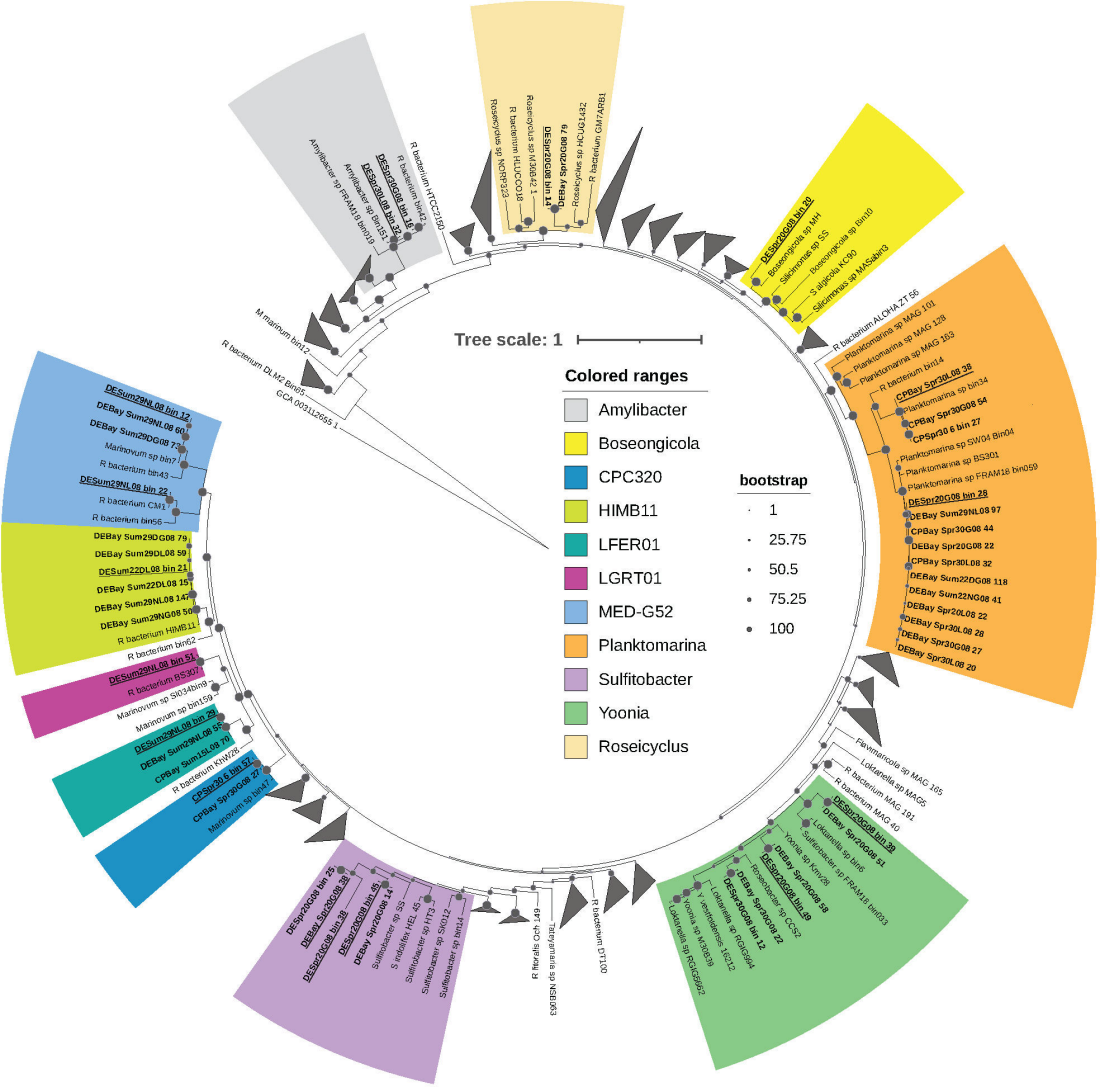
Phylogenomics of *Rhodobacterales* MAGs from the Delaware and Chesapeake Bays. A maximum likelihood tree of 28 concatenated single-copy ribosomal proteins derived using anvi’o from a total of 338 *Rhodobacterales* MAGs/genomes from this study and others (33, 34). The RAxML program was used to obtain the tree with the PROTGAMMALG model of rate heterogeneity (32). The tree was annotated in iTOL (35). Bootstrap values of 70% or higher from 1,000 sample trees were represented through dark circles on the nodes. A *Cyanobacteria* sp. was used to root the tree. Multiple genomes in clades without any MAG from this study were collapsed together into gray wedges. Bold text indicates MAGs from this study, and dereplicated ones are highlighted with underscores. Eleven genera to which MAGs from this study belonged were highlighted with colors. Abbreviations: CP = Chesapeake Bay; DE = Delaware Bay; Spr = Spring; Sum = Summer; the numbers following bay name and season indicate salinity in PSU; D = day; *N* = night; G08 = >0.8 µm, and L08 = <0.8 µm size fraction.

From the 36 assembled metagenomes, 46 MAGs with >80% completeness and <5% contamination were taxonomically classified using GTDB-Tk as members of the order *Rhodobacterales* (31). The average size of the medium- to high-quality MAGs was 2.8 ± 0.53 Mbp and the average GC content was 52.6% ± 5.5% (Data Set S1). Both GTDB-Tk-based taxonomic affiliation and position in a phylogenomic tree indicated that these MAGs belonged to 11 *Rhodobacterales* genera, namely, *Amylibacter* (Am), *Boseongicola* (Bo), CPC320 (CP), HIMB11 (HI), LFER01 (LF), LGRT01 (LG), MED-G52 (ME), *Planktomarina* (Pl), *Roseicyclus* (Ro), *Sulfitobacter* (Su), and *Yoonia* (Yo) (Fig. 1) (31, 32). Abbreviated genus names were added to the representative MAG names for clarity. Each genus contained at least one MAG from this study with one or more *Rhodobacterales* genomes, MAGs, or single amplified genomes (SAGs) from other studies. Among the Chesapeake and Delaware Bay MAGs, 14 were related to *Planktomarina*, 6 to HIMB11 or *Yoonia*, 5 to *Sulfitobacter*, 4 to MED-G52, 3 to LFER01, 2 each were related to *Amylibacter*, CPC320 or *Roseicyclus*, and only one each was related to *Boseongicola* and LGRT01 (Fig. 1; Data Set S1). After dereplication at a 95% average nucleotide identity (ANI) cutoff to find representative genomospecies, *Sulfitobacter* and *Yoonia* each had three representative species; *Amylibacter*, MED-G52, and *Planktomarina* genera each had two, while *Boseongicola*, CPC320, HIMB11, LFER01, LGER01, and *Roseicyclus* each had one (Data Set S1).

### *In situ* abundances

**Fig 2:**
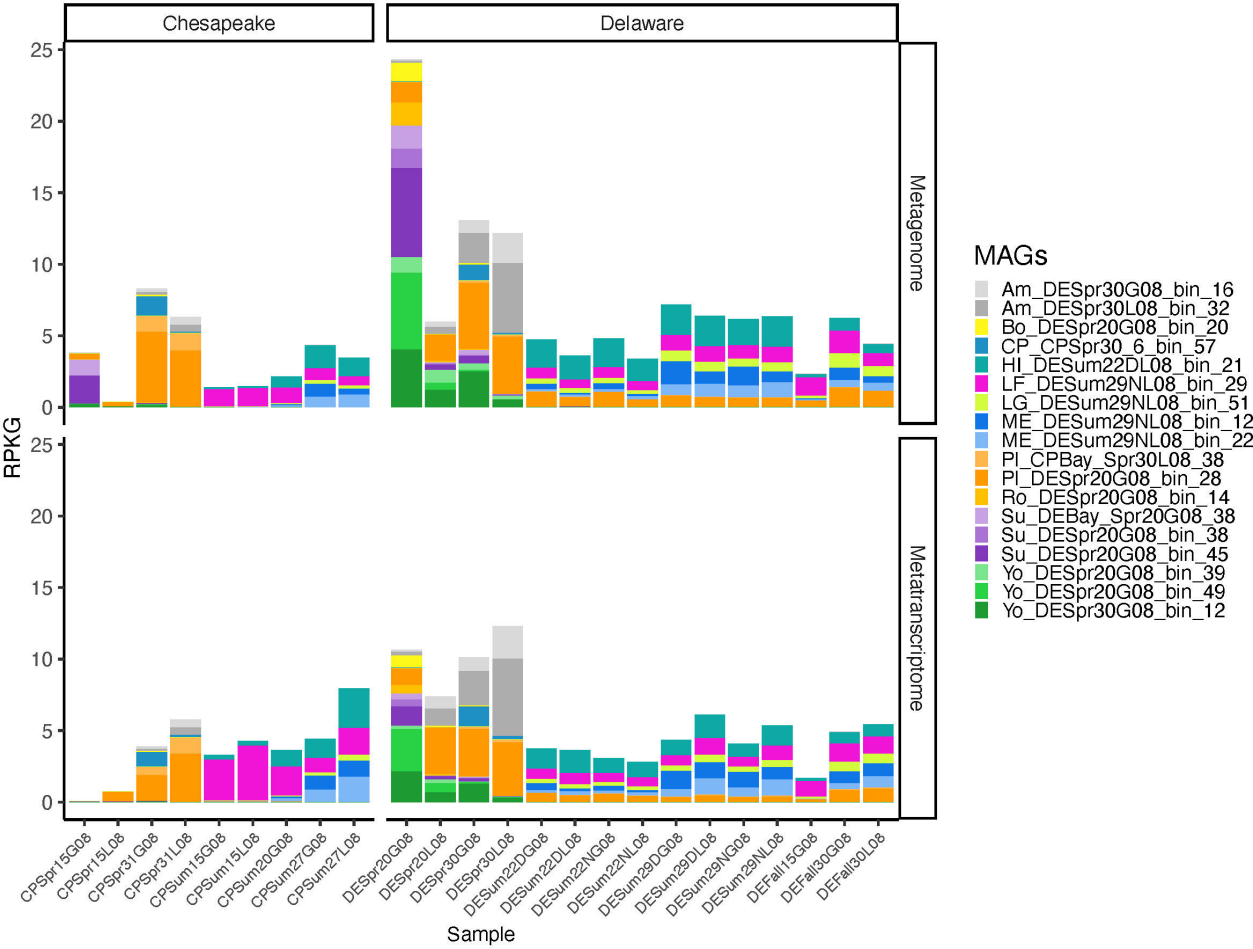
RPKG of *Rhodobacterales* in the Chesapeake and Delaware Bay metagenomes and metatranscriptomes. Abundance via RPKG values was calculated based on the featureCounts table and the total length of all ORFs in each representative MAG (38–40). Abbreviations: CP = Chesapeake Bay; DE = Delaware Bay; Spr = Spring; Sum = Summer; the numbers following indicate salinity in PSU; D = day; *N* = night; G08 = >0.8 µm, and L08 = <0.8 µm size fraction. Two letters at the beginning of a MAG indicate the genus to which it belongs according to GTDB-Tk taxonomy (31). Abbreviations: Am = *Amylibacter*; Bo = *Boseongicola*; CP = CPC320; HI = HIMB11; LF = LFER01; LG = LGRT01; ME = MED-G52; Pl = *Planktomarina*; Ro = *Roseicyclus*; Su = *Sulfitobacter*; Yo = *Yoonia*.

The *Rhodobacterales* MAGs assembled from the Chesapeake and Delaware Bay metagenomes represented between 0.1% and 12.2% or 0.4% and 24.3% of the entire community in samples as revealed by singleM or number of reads recruited per kilobase of MAG and gigabase of metagenome (RPKG) analyses, respectively (Fig. 2; Fig. S2) (36). Moreover, together these MAGs covered a significant portion (20.2%–4.8%) of all *Rhodobacterales* estimated through Kaiju read-based coverage analysis (Fig. S2) (37). Distinct patterns were observed in the RPKG profiles of the representative *Rhodobacterales* genomospecies, especially between salinities, seasons, and bays (Fig. 2). *Rhodobacterales* were not detected in low salinity samples but were abundant in all medium- and high-salinity samples. One *Planktomarina* genomospecies, Pl_DESpr20G08_bin_28, was especially abundant in both bays in spring and the Delaware Bay in summer while the other *Planktomarina* genomospecies, Pl_CPSpr30L08_38, was most abundant in high salinity Chesapeake Bay spring samples, along with two *Sulfitobacter* genomospecies. All *Yoonia*, *Sulfitobacter,* and *Roseicyclus* genomospecies were abundant in Delaware Bay spring samples. *Boseongicola* genomospecies were abundant in medium-salinity and *Amylibacter* genomospecies were abundant in high-salinity Delaware Bay spring samples. HIMB11-, LFER01-, LGRT01-, and MED-G52-related genomospecies were abundant in summer from both bays. Most genomospecies were equally represented in the metagenomes and metatranscriptomes except the LFER01-related genomospecies, which had higher abundances in the metatranscriptomes than in metagenomes in the Chesapeake Bay summer samples.

Spearman correlation analysis between environmental variables and a Mantel test revealed that the average abundance, expressed as RPKG, of representative *Rhodobacterales* MAGs from these bays was significantly and positively correlated (*P* < 0.05) with salinity, temperature, and phosphate and silicate concentrations from summer samples (Fig. S3a) (41). However, a similar analysis was not done for spring due to the low number of samples. When analyzed individually, the abundance of *Planktomarina*, HIMB11, LFER01, and MED-G52 genomospecies was all positively correlated with temperature (*P* < 0.05) and silicate concentrations (*P* < 0.1), all but *Planktomarina* were positively correlated with phosphate concentrations (*P* < 0.05) and two (*Planktomarina* and MED-G52) were positively correlated with cell density (*P* < 0.05) (Fig. S3b and c).

### Pangenome analyses

**Fig 3:**
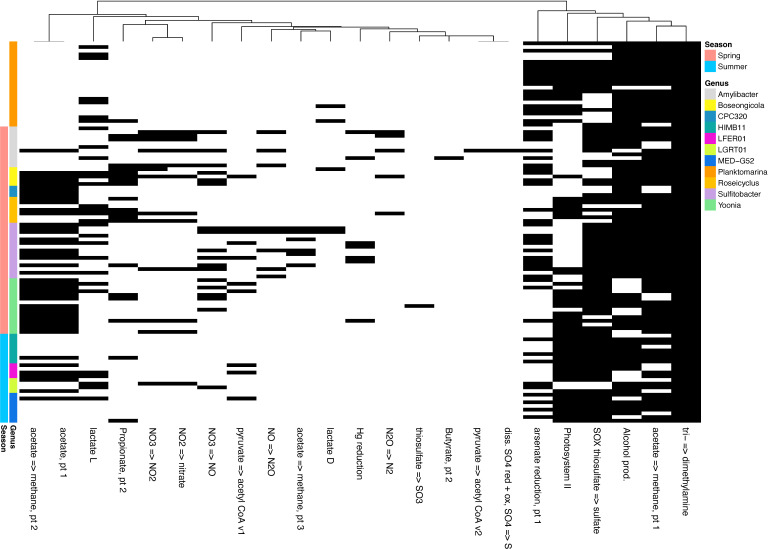
Pangenome analysis of 11 selected *Rhodobacterales* genera. In all members of 11 genera with representatives found in the Chesapeake and Delaware Bays, genes were annotated using DRAM (42), and the presence of key genes of select metabolic pathways is shown. The color bar on the left indicates the different genera and their seasonal dominance.

We performed pangenome analysis to determine the functional potentials of the 11 *Rhodobacterales* genera above, including all 46 MAGs recovered from the Chesapeake and Delaware Bay samples and 57 previously reported genomes/MAGs (31, 33, 42). Each of the 11 genera had more than 9 members on average, though CPC320 had only three and *Planktomarina* had 23 members (Data Set S2). Genes found in all genera featured full pathways for glycolysis (Embden-Meyerhof pathway), pentose phosphate, citrate (TCA or Krebs), and glyoxylate cycles. Moreover, several electron transport chain complexes (I–V), associated with aerobic respiration, were found to be fully covered in the pangenome. Among the common metabolic processes encoded by genes in most *Rhodobacterales* genera found in these bays were C1 metabolism, alcohol production, thiosulphate oxidation to sulfate through the sulfur oxidation (Sox) enzyme system, and the light reactions in photosynthesis (Fig. 3). Genes related to nitrogen-transforming processes—such as nitrogen fixation, nitrification, denitrification, and dissimilatory nitrate reduction to ammonium were not common in the pangenome.

### Differences in metabolic pathways

*Rhodobacterales* genera differed in their metabolic potentials on many occasions. For instance, all members of CPC320, HIMB11, LFER01, MED-G52, and *Planktomarina* lacked genes associated with nitrogen-transforming processes. Among the spring dominant genera, *Amylibacter* uniquely possessed the ability to break butyrate, convert pyruvate to acetyl CoA, and transform sulfate to sulfide through the dissimilatory reduction process. Unlike the other genera, both *Amylibacter* and *Sulfitobacter* lacked genes for light reactions for aerobic anoxygenic phototrophy (AAP). *Sulfitobacter* were among the few with the potential to use polyphenolics. *Yoonia* uniquely had genes involved in the metabolism of xyloglucan and sulf-polysaccharides and the oxidation of thiosulfate to sulfite (Fig. 3; Data Set S2).

### Transporters

Transporter genes were among the most abundant in all members of the pangenome. We identified 38 transporter genes that were present in >50% of members of each genus, and ~13% of such genes were found in all MAGs (Data Set S2). Such common genes included *livFH*, ABC.PE.P, and ABC.FE.SP for branched-chain amino acid, peptides/nickel transport, and iron transport systems, respectively. Some transporter genes were uniquely present or absent in a genus (Fig. S4). For instance, *glpPQV*, and *rhaPS* for glycerol, and rhamnose transport, respectively, were found in all members of CPC320. Genes for alpha-1,4-digalacturonate and putative thiamine transport systems were uniquely observed in >75% of members of *Roseicyclus* and *Yoonia*, respectively. Though common in nine genera, *Sulfitobacter* and *Yoonia* lacked the *mlaC* gene for phospholipid transport system.

### CAZymes

The differential presence of carbohydrate-active enzymes (CAZymes) was assessed in the pangenome, as carbohydrates from various sources provide energy for estuarine microbes, especially for PA bacteria (43). The number of CAZy genes in representative *Rhodobacterales* MAGs ranged from 1 to 50 and they belonged to 53 CAZy subfamilies (Fig. S5) (44–46). *Sulfitobacter*, *Roseicyclus*, *Yoonia*, and *Planktomarina* MAGs contained a variety of CAZymes. However, representative MAGs of *Amylibacter*, HIMB11, LFER01, and MED-G52 were less versatile than others in regard to the types and numbers of CAZymes they contained. Sub-families AA3_2 (auxiliary activities), GH23 (glycoside hydrolases), GT2, GT4, GT19, and GT28 (glycosyl transferases) were present in about 80% of the analyzed genera. The number of GT2 and GT4 genes was much higher in the *Roseicyclus* and *Sulfitobacter* representative MAGs compared to the others.

### *In situ* growth estimates

**Fig 4:**
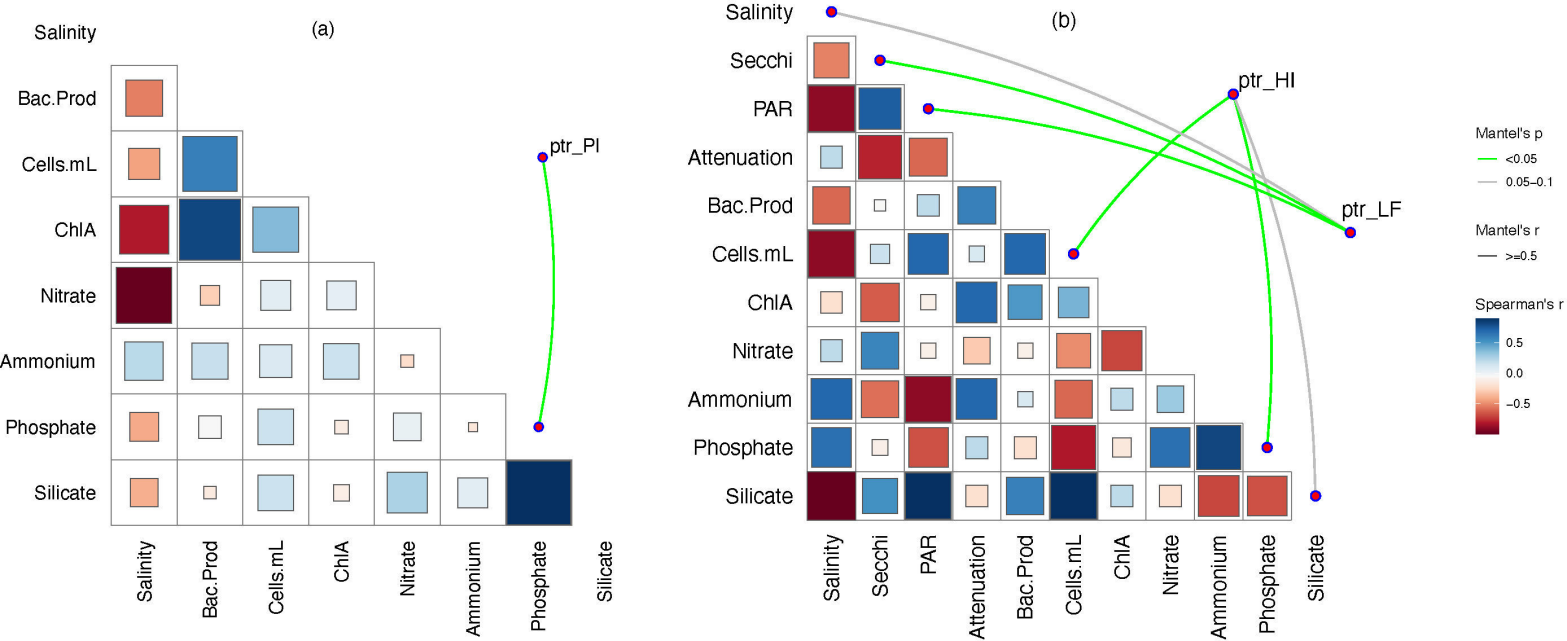
Relationships between estimated growth rates of *Rhodobacterales* and environmental factors. log_2_PTR values of genomospecies and related environmental parameters of samples were analyzed for Mantel’s correlation and *P*-values using the vegan package in RStudio (47, 48). (**a**) *Planktomarina* (ptr_Pl) and (**b**) HIMB11 (ptr_HI) and LFER01 (ptr_LF).

Analyzing the true growth rates of *Rhodobacterales* members in these two bays was beyond the scope of the study. However, peak-to-trough ratio (PTR)-based analyses (47) of representative *Rhodobacterales* genomospecies MAGs from our data set indicated differences in estimated growth rates, which varied among taxa (Table 1) and depended on environmental conditions. A significant positive correlation (*P* < 0.05) was found between PTRs of *Planktomarina*, HIMB11, LFER01 genomospecies, and various combinations of light availability, cell density, and phosphate concentrations (Fig. 4). *Planktomarina* and HIMB11 growth rate estimates were correlated with phosphate concentrations (*P* < 0.05), HIMB11 growth rate was also correlated with cell numbers (*P* < 0.05) and weakly with silicate (*P* < 0.1) while LFER01 growth rate was correlated with light (PAR and Secchi) and weakly with salinity.

**TABLE 1:** Estimated growth rates of *Rhodobacterales* in the Chesapeake and Delaware Bays^*a*^

			*Amylibacter*		CPC320	HIMB11	LFER01	MED-G52		*Planktomarina*		*Sulfitobacter*		*Yoonia*
Bay	Season	PSU-SF	Am_16	Am_32	CP_57	HI_21	LF_ 29	ME_12	ME_22	Pl_38	Pl_28	Su_38	Su_45	Yo_12
CP	Spr	15G08									0.84	0.62	0.79	
		31G08		1.03	0.62					0.57	0.59			0.49
		31L08	1.29	0.89	0.56					0.64	0.65			
	Sum	15L08				0.54	0.58							
		20G08				0.64	0.53							
		27G08				0.73	0.49	0.68	0.55					
		27L08				0.69	0.60	0.63	0.65					
DE	Spr	20G08		0.81	0.51						0.52	0.55	0.50	0.69
		20L08	1.01	0.85							0.44		0.33	0.85
		30G08	1.00	0.84	0.63						0.56	0.52	0.52	0.68
		30L08	1.11	0.81	0.48					0.54	0.49			0.74
	Sum	22DG08				0.73	0.54	0.64	0.37		0.54			
		22DL08				0.49	0.39	0.46	0.36		0.36			
		22NG08				0.69	0.54	0.61	0.45		0.48			
		22NL08				0.69	0.52	0.63	0.40		0.42			
		29DG08				0.73	0.50	0.74	0.58		0.54			
		29DL08				0.55	0.47	0.64	0.49		0.36			
		29NG08				0.73	0.49	0.69	0.54		0.52			
		29NL08				0.55	0.43	0.64	0.47		0.37			
	Fall	15G08				0.52	0.36				0.37			
		15L08				0.56	0.41				0.40			
		30G08				0.55	0.30	0.62	0.49		0.48			
		30L08				0.49	0.29	0.56	0.39		0.32			

^*a*^ PSU-SF: Salinity in PSU- Size fraction; CP = Chesapeake Bay; DE = Delaware Bay; Spr = Spring; Sum = Summer; D = day; N = night; abbreviated MAG names are mentioned in Data Set S2.

Among all representative genomospecies, estimated growth rates in the spring were the highest in the *Amylibacter* MAG, ranging from 0.81 to 1.3 while those from CPC-320, *Sulfitobacter*, and *Yoonia*-related MAGs ranged from 0.33 to 0.85 (Table 1). For Pl_DESpr20G08_bin_28*,* growth rates averaged 0.59 ± 0.12 in both bays in spring and less in Delaware Bay summer samples, averaging 0.45 ± 0.08. Growth rates from representative HIMB11, LFER01, and MED-G52 MAGs averaged 0.29–0.74 in summer. Overall, the summer genomospecies had higher average growth rates in the Chesapeake (0.55–0.69) compared to the Delaware Bay (0.44–0.58). Estimated growth rates in the large size fraction were significantly higher (*P* < 0.05) than the small size fraction for Pl_DESpr20G08_bin_28 and HI_DESum22DL08_bin_21 and ranged from 0.37 to 0.59 and 0.51–0.73, respectively, in the large size fraction vs. 0.32–0.65 and 0.49–0.69, respectively, in the FL fraction. There were no significant differences in estimated growth rates between day and night for any of the MAGs. The codon usage bias-based program, gRodon (49), was also used to estimate the maximum growth rates of MAGs and indicated that two MED-G52 MAGs had the longest maximal doubling time of >9 h, whereas HIMB11- and *Sulfitobacter-*related MAGs had the shortest, with maximum doubling times of <2 h (Table 1; Data Set S1).

### *Rhodobacterales* gene expression

**Fig 5:**
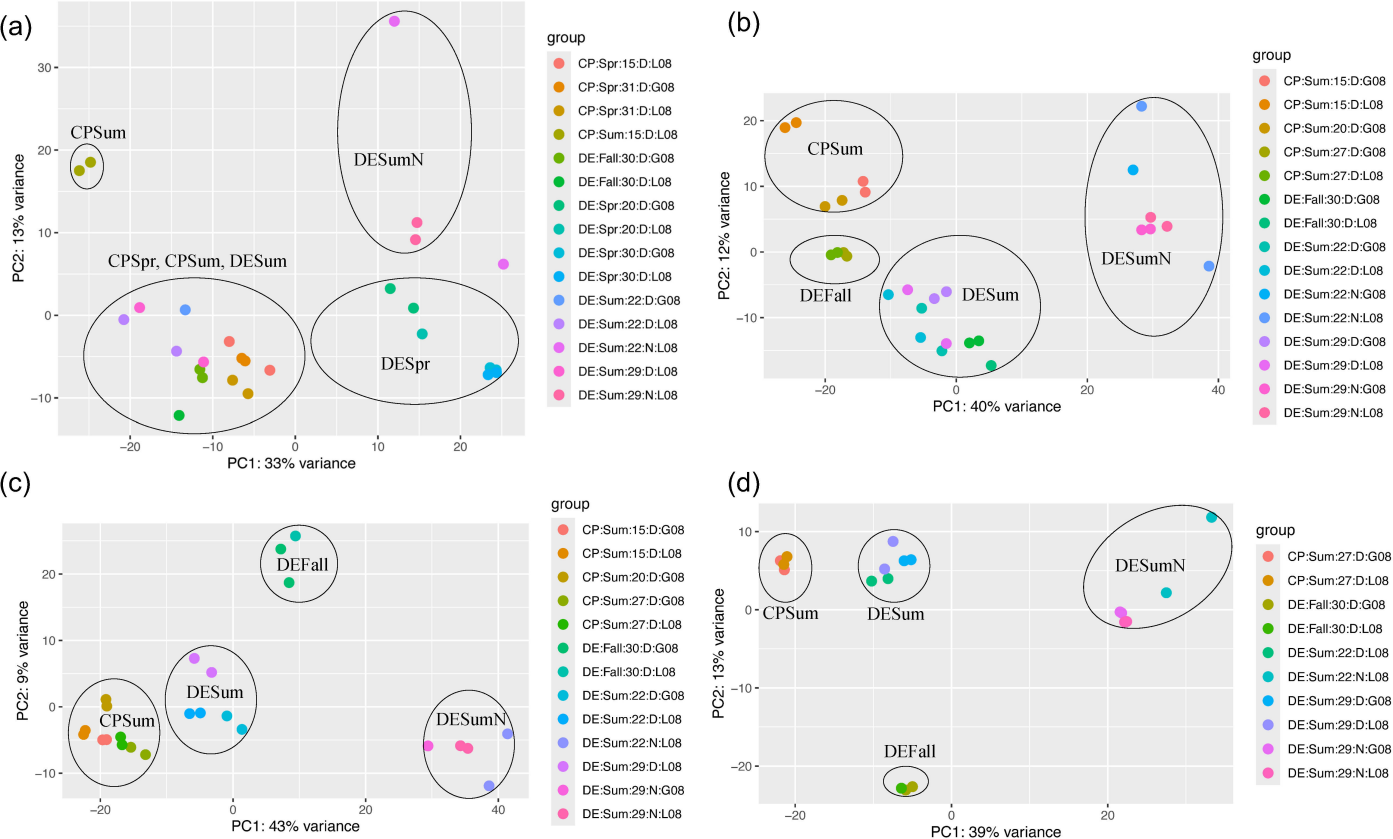
Ordination plots of gene transcripts from four abundant *Rhodobacterales* MAGs. PCA plot of DESeq2 normalized and transformed transcript abundances of representative MAGs from (**a**) *Planktomarina*, (**b**) HIMB11, (**c**) LFER01, and (**d**) MED-G52 in different samples, corresponding to different environmental conditions (50). Clusters were circled and labeled manually for visual comparison. Abbreviations: CP = Chesapeake Bay; DE = Delaware Bay; Spr = Spring; Sum = Summer; the numbers following bay name and season indicate salinity in PSU; D = day; *N* = night; G08 = >0.8 µm, and L08 = <0.8 µm size fraction.

We hypothesized that *Rhodobacterales* activities, as estimated by their gene expression, would reflect their relative abundance and estimated growth rates depending on environmental conditions. RNA-Seq data of four MAGs with relatively high abundance and estimated growth rates in multiple samples (HI_DESum22DL08_bin_21, ME_DESum29NL08_bin_12, Pl_DESpr20G08_bin_28, and LF_DESum29NL08_bin_29) were analyzed from medium- and high-salinity metatranscriptome samples. The four genomospecies had distinct gene expression patterns that varied with environmental conditions, such as time of day, location (bay), season, and size fraction (Fig. 4). Significant differences in gene expression of all four genomospecies were observed between night and day samples. Other noticeable differences occurred between bays and seasons. Heatmaps of the top 50 highly expressed genes indicated common trends in activity between these MAGs (Fig. 5; Fig. S6). Transporters were the most highly expressed genes in all four MAGs. The largest differences in the number of differentially expressed genes between conditions was between bays, with an average of 8.6% and 7.6% of aggregated genes differentially expressed in the summer PA or FL size fraction, respectively, in three of the four MAGs (Table S1). Gene expression in the *Planktomarina*-related genomospecies was the least affected by all environmental conditions and the LFER01-related one was the most, with an average of 4.3% and 9.9% differentially expressed genes, respectively.

### Gene expression differences between night and day

Daylight was the most common environmental factor influencing changes in gene expressions of the investigated *Rhodobacterales*. On average, 6.4% ± 2.7% of the total genes from these four MAGs were differentially expressed between night and day in both bays combined (Fig. 5; Table S1). All or a subset of genes from *pufABCLM* encoding reaction center proteins for AAP were expressed highly at night compared to the day in all four MAGs we tested in the Delaware Bay summer samples (Fig. 5; Fig. S6). In the HIMB11-related MAG, *bchXY* genes encoding bacteriochlorophyllide reductase subunits were also expressed more at night than during the day (Table S1). On the other hand, *coxLMS* genes encoding carbon monoxide dehydrogenase were expressed more in all four MAGs in the day compared to night samples. In addition, in the HIMB11-related MAG, genes encoding the tripartite tricarboxylate transporter family proteins TctA and TctB were expressed more during the day compared to night. Similar trends in transporter gene expression between night and day were seen in the other three MAGs.

### Gene expression differences between the two bays

*Rhodobacterales* MAGs differed in the expression of certain genes between the two estuaries under the same conditions. In summer, the HIMB11 genomospecies had many genes like *tctAC*, *dctM*, *fadL*, *livHK,* and *yiaO* encoding transporter proteins of tripartite tricarboxylate, C4-decarboxylate, branched-chain amino acids, long-chain fatty acids, and TRAP-type transport system periplasmic proteins, respectively, significantly less expressed in the medium salinity FL size fraction samples of the Chesapeake compared to the Delaware Bay (Data Set S3). By contrast, expression of genes such as *rplBEMNQSUVX* and *rpsAGKLMNOQU*, responsible for encoding the large and small ribosomal subunits, respectively, were higher in the Chesapeake compared to Delaware under identical conditions. Furthermore, genes including *argF* and *acpP*, which encode anabolic enzymes for arginine and various secondary metabolite biosynthesis, respectively, showed higher expression levels in the Chesapeake than in the Delaware Bay. In the LFER01-related MAG, a trend of significantly higher expression of genes encoding ribosomal proteins (*rplBKMNOSUXY* and *rpsADGLMT*) in summer medium salinity FL samples from the Chesapeake compared to the Delaware Bay was observed. Moreover, expression of genes like *livK*, *tctC*, *yiaO*, and *proWX* encoding various transporters was lower in the Chesapeake than the Delaware Bay. In the MED-G52 genomospecies, only *rpsU* gene expression was significantly higher in high-salinity PA samples in the Chesapeake than in the Delaware Bay during summer. However, expression of genes like *tct*, *yia*, *liv,* and *pro* encoding various transporters was less in the Chesapeake than the Delaware Bay under the same conditions. For the *Planktomarina*-related genomospecies, differential ribosomal protein expression in the Chesapeake than the Delaware Bay was mostly insignificant except for the *rplM* gene which had higher expression in the former than the latter during spring. However, gene expression of transporters was significantly higher in the Delaware than in the Chesapeake Bay in the spring.

### Other gene expression differences

**Fig 6:**
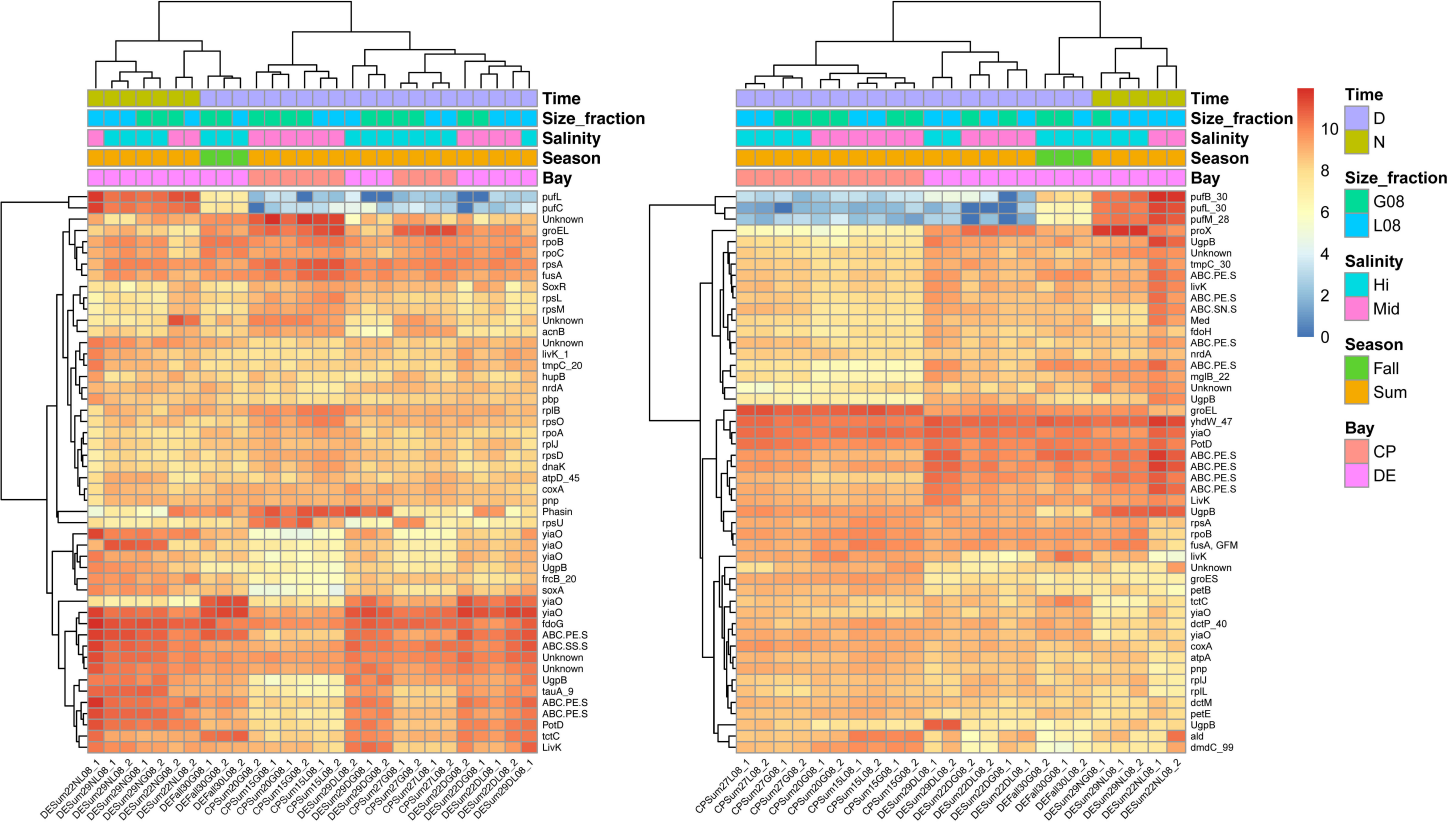
Top 50 most highly expressed genes of (**left**) HI_DESum22DL08_bin_21 and (**right**) LF_DESum29NL08_bin_29 in varying conditions. The most highly expressed 50 genes of these genomospecies were identified using DESeq2 (50). Abbreviations: CP = Chesapeake Bay; DE = Delaware Bay; Sum = Summer; # =salinity in PSU; G08 = >0.8 µm and L08 = <0.8 µm size fractions; # =RNA1 or RNA2; D = Day; *N* = Night. The gene symbol key is noted in the supplemental material.

Gene expression also varied with season, salinity, or size fraction (Data Set S3; Fig. 5 and Fig. 6; Fig. S6). For the HIMB11-related MAG, genes encoding tripartite-type tricarboxylate transporter TctC and (R)-benzylsuccinyl-CoA dehydrogenase were more highly expressed in summer compared to fall. In *Planktomarina*, transporter proteins were expressed more in spring than the fall (Data Set S3). In addition, *bchXY* genes and others involved in light harvesting were expressed in the *Planktomarina*-related MAG more in the fall compared to the summer. In spring, *alkB* for alpha-ketoglutarate-dependent dioxygenase and a pectate lyase superfamily gene and many genes for proteins of uncharacterized functions were expressed more compared to fall in this same MAG.

Only a few genes were differentially expressed between salinities or bacterial size fractions (Table S1). In the *Planktomarina*-related MAG, genes encoding proteins for dimethylsulfoniopropionate (DMSP) degradation (DmdA), the phosphate transport protein (SphX), and L-cystate sulfo-lyase (CuvA) had higher expression in medium- compared to high-salinity spring samples in the Chesapeake Bay. On the other hand, genes such as *sugB* and *malK* for trehalose transport system permease and maltose/maltodextrin import ATP-binding protein, respectively, were more expressed in spring high salinities compared to medium salinities. In the HIMB11-related MAG, *rpmGH* and *rpsU* genes for the large and small ribosomal subunits, respectively, were expressed more in the PA compared to the FL size fraction in the Chesapeake Bay during summer. However, *rplCENQSU* and *rpsALO* for large and small ribosomal subunits, respectively, were expressed more in the small size fraction than the large fraction. The expression of genes encoding transporters was not significantly different between the two size fractions in the Chesapeake Bay.

## DISCUSSION

The *Rhodobacterales* are a versatile aquatic bacterial order and can comprise up to a quarter of the total marine bacterioplankton (6, 10, 51). They are chemoheterotrophs and many can utilize light, sulfur, and CO for additional energy (6, 52). In the current study, we identified 18 *Rhodobacterales* genomospecies within 11 genera which varied in their metabolic and environmental niches as evidenced by genomic, abundance, estimated growth rate, and activity differences. In the Delaware Bay, *Rhodobacterales* relative abundance (as reflected by RPKG) in our study was up to 24% which was close to ~20% found previously (27). Many genomospecies inhabited the same environment, were relatively equal in their abundance, and had similar overall metabolisms. However, most contained specific substrate-utilization and energy-production potentials and activities that varied with season, nutrient concentrations, or time of day.

### Wide metabolic potentials of *Rhodobacterales* from the Delaware and Chesapeake Bays

Roseobacters have been considered “opportunitrophs” adapting to various lifestyles with their flexible genomes (53). As with other studies, most of the *Rhodobacterales* genomospecies found in this study could be considered specialists adapted better to a specific season and salinity as seen with other taxa (54, 55). However, a sharp delineation between generalist and specialist *Rhodobacterales* may be unclear, especially during blooms when both types are active (56, 57). In agreement with this, the *Rhodobacterales* in these bays exhibited a wide range of metabolic potentials, indicating their diverse roles in biogeochemical cycling, with many genes, especially transport-related ones, present in only a subset of the examined genera. However, the *Planktomarina* genomospecies Pl_DESpr20G08_bin_28 was seen in all seasons and both bays in mid and high salinities. Its estimated growth rate did not vary much, nor did the percentage of differentially expressed genes between various conditions. These combined observations support the notion that *Planktomarina* can potentially adopt a generalist lifestyle (55).

### Multiple energy production mechanisms of *Rhodobacterales* in eutrophic ecosystems

*Rhodobacterales* contained several energy production mechanisms in these two eutrophic estuarine ecosystems. Nine out of eleven genera analyzed from these two bays contained genes necessary to use CO, folate, thiosulfate, and light for energy production, indicating their lithotrophic potential. To provide retention of organic carbon in marine food webs, the prevalence of *cox* genes in Roseobacters is almost universal and well documented from studies by Moran et al., Newton et al., and Cunliff et al. (13, 58, 59). Moreover, folate biosynthesis of *Dinoroseobacter shibae* was reported as an integral part of symbiosis with the alga *Ostreococcus tauri* underlining the importance of this metabolic ability (60). Roseobacters are well known for their ability to use sulfur and dimethyl sulfur compounds for energy production, especially after phytoplankton blooms, influencing the global sulfur cycle (2, 12, 61). Thiosulfate oxidase and sulfite dehydrogenase encoding genes found in most genomospecies were expressed more in high- compared to low- and mid-salinity samples in both bays, indicating the importance of sulfur-based lithotrophy in higher salinity waters. Similar to another study, the *dmdA* gene for the DMSP cleavage pathway was expressed by LFER01, HIMB11, and *Planktomarina* in both bays (62). Almost all *Rhodobacterales* from our study, except the genera *Amylibacter*, *Boseongicola,* and *Sulfitobacter,* would be considered AAP bacteria, as they contained and expressed genes for acquiring light energy through AAP. AAP genes were also commonly found in other Chesapeake and Delaware Bay studies (63, 64). Preheim et al. found many species contained *pufL* and *pufM*, and *Planktomarina* was the most common among them (63). While no significant differences in growth rates were observed between day and night, the nighttime expression of AAP genes may represent a preparatory strategy for diurnal energy harvesting (65, 66).

### Relative abundance and estimated growth rates

A combination of estimated growth rates plus abundance provided a better understanding of environmental effects on the presence and activities of this taxonomic order than the latter alone, which agrees with other studies (27, 47, 67, 68). As there were no significant difference in growth rates between day and night, they probably do not maintain a circadian rhythm like Cyanobacteria (69). Their estimated growth rates may be related to genome size and lifestyle; small, FL, oligotrophic generalists are more apt to be slow growing than larger, PA copiotrophs with more specialized metabolic strategies (70). Both HIMB11 and *Planktomarina* genomospecies had higher estimated growth rates in the PA compared to the FL fraction. This suggests their ability to use nutrients from particles or larger cells such as phytoplankton for biomass production.

*Rhodobacterales* abundance and estimated growth rates likely have some shared control mechanisms, as total and individual *Rhodobacterales* abundances and estimated replication rates correlated with phosphate and silicate concentrations from both bays. *Rhodobacterales* was found among the efficient dissolved organic phosphorus hydrolyzers using alkaline phosphatase in the Northwestern Mediterranean Sea (71, 72). Previous studies found silicate a key component supporting diatom blooms which likely provide organic carbon and nitrogen to various *Rhodobacterales* allowing for fast growth (56, 73). A *Rhodobacterales*, *Ruegeria pomeroyi* DSS-3, expressed more transporters to get various amino acids, nucleosides, and organic sulfur compounds from exometabolites in a microcosm with the diatom, *Thalassiosira pseudonana* (74). Previously, salinity was reported as the key factor in controlling marine bacterioplankton (27) and we also found it important for the average abundance of *Rhodobacterales*, especially *Planktomarina* and MED-G52. The RCA (*Roseobacter* clade affiliated) cluster abundance in the North Sea was found inversely correlated with salinity which ranged there from ~28 to 35 PSU, which we did not observe in our study covering freshwater to marine salinities. We did not find medium- or high-quality *Rhodobacterales* MAGs in low-salinity samples, possibly due to excessive species heterogeneity (51).

Environmental factors affecting *Rhodobacterales* estimated growth rates varied among the tested genomospecies. For example, estimated growth rates of both *Planktomarina* and HIMB11 were correlated with phosphate concentrations indicating their higher replication in phosphate-rich environments. The estimated growth rate of LFER01 was affected by PAR and Secchi disk depth (light availability) indicating that an increasing light promoted photoheterotrophy in this strain. Other environmental factors not measured here, such as carbon and sulfur concentrations and types, may also help explain differences in abundance vs. estimated growth rates.

### Ubiquity and activity of *Planktomarina* in these estuaries

*Planktomarina* genomospecies were ubiquitous in both bays and in the spring, summer, and fall in Delaware bay, specifically with a higher abundance of the *Planktomarina*-related MAG Pl_DESpr20G08_bin_28 in spring compared to summer. In Delaware Bay, *Planktomarina* estimated growth rates and transcript abundances were higher in spring than fall and may be related to daylight differences during the sampling time of 07:00 hours. These might indicate the efficiency of this genomospecies in using available resources in different seasons and locations. Previously, *Planktomarina* were reported to thrive in various marine ecosystems, successfully interacting with flora and fauna and producing energy using AAP, sulfur, and CO metabolisms (55, 75). Here, a *Planktomarina*-related MAG from the spring had higher growth rates in the PA than the FL fractions, and this agreed with other studies where some PA bacteria were found to be more active than many FL bacteria in estuaries (27). However, *Planktomarina* MAGs and amplicon sequence variants (ASVs) were less abundant in PA fraction than the FL fraction in samples collected from the southern North Sea and the Southern Ocean, respectively (76, 77). Our results also indicate differences in gene expression of important PA-associated functions in *Planktomarina*. In Delaware Bay, highly expressed genes during summer were in the ABC-type xylose transport system, followed by TRAP transporters and these gene products might help in acquiring bloom-associated metabolites. Moreover, high expression of the *livK* gene for the transport of branched-chain amino acid there suggests their competitive advantage when attached to larger particles to get low-molecular-weight substrates (78). Also, higher expression of the pectate lyase gene in spring compared to fall suggested more need for this enzyme to degrade pectin, a major cell wall component of eukaryotic phytoplankton and to help colonize particles containing mixed polysaccharide pools (79). The *Planktomarina*-related MAG expressed more ABC-type transporters likely acting as osmoprotectants (80) in high- compared to medium-salinity samples. In addition, phosphate transporters and AAP genes were expressed significantly more in medium than high salinity, and sugar transporters and DMSP demethylase were expressed more in high compared to medium salinities. These protein products likely help the adaptation of *Planktomarina* to rapidly changing estuarine environments.

### *Rhodobacterales* abundance and activity in the spring

The abundance and estimated growth rate patterns of *Rhodobacterales* were distinct in both bays, depending on the season. In spring, *Amylibacter*, *Sulfitobacter,* and *Yoonia* were highly abundant and had relatively high estimated rates of replication, especially in Delaware Bay. Overall, these spring genera had similar metabolic potentials, especially regarding sulfur, nitrogen, and C1 metabolism. In addition, they had higher numbers and diversities of CAZymes than other genera which enable them to use bloom-derived carbohydrates. These *Rhodobacterales* possessed genes for phosphate and sulfate transporters, likely enabling them to use eukaryotic bloom-derived organic phosphates and sulfates (81). Also, their activity was likely controlled by eukaryotic phytoplankton blooms prevalent during that season, as evidenced by the highest level of chlorophyll *a* in the mid-salinity Delaware Bay spring sample compared to other samples as well as with historically measured phytoplankton blooms in the spring (29, 82). Previously, FL *Amylibacter* was found associated with spring phytoplankton blooms and after the first bloom responders declined (76, 77). Moreover, they were reported to be involved in DMSP degradation and also in the production of cobalamin supporting microbial plankton growth (83, 84). Also, A*mylibacter* was previously found attached to particles and bloomed with increased DOM availability (59, 85, 86). *Yoonia-Loktanella* and *Sulfitobacter* were abundant and active with organic matter induction by a phytoplankton bloom in the surface water of the Weddel Sea of the Antarctic Southern Ocean (87).

### *Rhodobacterales* abundance and activity in the summer

HIMB11-, LFER01-, and MED-G52-related genomospecies showed diverse metabolic potentials and activities and were among the most abundant with rapid estimated growth in summer, especially in the Chesapeake Bay. Phosphate concentrations influencing the abundance of HIMB11, LFER01 and MED-G52 suggested that phosphate availability plays a key role in their abundance in these estuaries. The positive correlation of HIMB abundance and silicate concentrations indicated increased diatom presence in the Chesapeake Bay in summer to might enhance this genus (82, 88). Estimated growth rates of HIMB11 were correlated with phosphate concentrations and cell density, also indicating their enhanced ability to replicate during algal blooms. This aligns with previous reports identifying HIMB11 as one of the most common and opportunistic Roseobacters, particularly in summer (89–91). We also observed that the HIMB11-related MAG had higher estimated growth rates in the PA than the FL fractions. HIMB11 was also found abundant and proliferating in the PA fraction from Daya Bay in Southern China (92). Here, the HIMB11-related genomospecies showed significantly higher expression of genes for ribosomal proteins and lower expression of genes for various transporter proteins which supported their higher growth rate estimates in the Chesapeake than the Delaware Bay. In nutritionally or energy-favorable conditions, these genomospecies likely maintained high ribosome and translational machinery concentrations for rapid growth and avoided associated physiological constraints on gene expression by reducing excess levels of certain proteins (like transporters) (93). In addition, nitrogen regulation proteins and ammonium transporters were expressed more in the Chesapeake than the Delaware Bay, indicating the need for N compounds during rapid growth.

LFER01 and MED-G52 genera are less characterized compared to HIMB11 and have only recently been reported (94, 95). MED-G52 could not be analyzed much due to insufficient abundances but we found positive correlations between estimated LFER01 growth rates and light availability in summer when there is high irradiance. In addition, the representative LFER01 genomospecies was abundant in the metatranscriptomes and had highly expressed genes like *coxL* for energy conservation, and *proX* and *livK* for amino acid transport in summer in the Delaware Bay compared to the Chesapeake Bay but had lower expression of ribosomal proteins which reflected their reduced estimated growth rates. Unlike our study, LFER01 species were seen to significantly affect the oxidation of CO to CO_2_ in spring in the Blanes Bay Microbial Observatory near the NW Mediterranean coast (96). LFER01 also showed high cell-specific respiration rates among other genera analyzed in the coastal Gulf of Maine (97). Unlike HIMB11- and LFER01-related genomospecies, changes in gene expression patterns between conditions for ribosomal or other proteins were not observed in MED-G52. However, MED-G52 highly expressed *fdh*/*fdo* genes for formate dehydrogenase are involved in C1 metabolism and may be important for their high abundance and growth rates in summer as previously reported (98).

### Conclusion

We identified both *Rhodobacterales* specialists and generalists in the two Mid-Atlantic estuaries, and their presence, activity, and estimated growth rates depended on environmental factors, especially temperature and nutrient availability. Our analysis encompassed well-studied members like *Planktomarina* and HIMB11, as well as lesser-discussed genera like *Boseongicola*, CPC320, and LFER01. Our findings revealed that estimates of growth rate correlated to transcript abundance differences, especially between two MAGs present in the summer, HIMB11 and LFER01, and that there were different controls like phosphate concentrations, evidence of diatom influence (through silicate concentrations), light, and cell density on abundances and estimated growth rates between these MAGs in the two estuaries. This diversity in niche adaptation underscores the importance of *Rhodobacterales* in biogeochemical cycling within estuarine and marine ecosystems. This knowledge and approach used will serve as a foundation for future in-depth analyses of specific taxonomic groups, and their contributions in dynamic estuarine environments.

## MATERIALS AND METHODS

### Sampling

Surface (~1.5 mbsf) water samples were collected during five cruises on the R/V Sharp using a rosette sampler with associated conductivity, temperature, and depth (CTD) profiles in 2014 and 2015 from the Delaware (DE) and Chesapeake Bays (CP), respectively. The samples were collected from these bays both seasonally and along a salinity gradient to vary environmental conditions to maximize differences between samples. This allowed us to determine major differences in taxonomic, functional potential, and activity/growth rates between different environmental conditions. A map showing the sampling sites was created using ArcGIS software by Esri (Fig. S1) (https://clemson.maps.arcgis.com/home/index.html) (99) . The water quality data were measured using a Sea-Bird data sonde, light intensity and attenuation were measured, and water samples were collected for nutrient concentrations and bacterial production measurements as previously reported (30, 100). Cell separation as large- and small-cell-size fractions by passing water samples sequentially through 0.8- and 0.22-μm-pore-size filters was described previously. The larger and smaller size fractions generally represented the PA and FL assemblages (29, 30). There were 12 metagenomes and 24 duplicating metatranscriptomes from points of the Chesapeake Bay. On the other hand, 24 metagenomes and 35 metatranscriptomes came from the Delaware Bay. Sample labels direct to the collection point and conditions as stated above. TruSeq library preparation kit (Illumina) was used for all metagenomes and metatranscriptomes to get sequences done by the Joint Genome Institute. Collections, extraction, and sequencing of metagenomic and duplicate metatranscriptomic samples as well as MAG generation were previously described (29, 30). Briefly, metagenomes were assembled using metaSPAdes and MAGs were binned using metaBAT2 (101, 102). Individual metagenomes were used for medium- and high-salinity samples. Pooled metagenomes were also tried for low-salinity samples where *Rhodobacterales* were generally absent.

### Quality assessment and taxonomic affiliation of *Rhodobacterales* MAGs

Quast v5.0.2 was used to find MAG characteristics like size, contig numbers, GC content, and N50 value (Data Set S1) (103). CheckM v1.1.3 (lineage_wf default parameters) revealed MAGs with >80% completeness and <5% contamination out of 46 *Rhodobacterales* MAGs from this study (104). Similarly, MAGs with >90% completeness and <10% contamination were added to the data set from a total of 1,753 MAGs found on the NCBI BioSample database when searched with keywords “Rhodobacterales” AND “metagenome” AND “marine” in March 2022. All genomes/MAGs were analyzed using GTDB-Tk v2.1.1 (classify_wf default parameters) to confirm their phylogeny (105). Dereplicated representative *Rhodobacterales* MAGs were obtained using dREP (v3.4.0) based on 95% ANI (106). Amino acid sequences of 28 single-copy ribosomal genes found in >80% of MAGs and genomes were concatenated and aligned using anvi’o v7.1; the alignment was trimmed with trimAl v1.4 to select reliable positions (33, 107). A snapshot maximum-likelihood tree was calculated in RAxMLv8.2.12 with LG substitution matrix and 1,000 bootstrap trees were used to establish support values (32). The tree was visualized and annotated in iTOL v5 to detail the phylogenetic position of the MAGs in relation to reported reference genomes (35). The ANI and average amino acid identity (AAI) between MAGs and reference genomes were calculated using the index_wf --fast -haai option of MiGA v0.7.26.2 (108) and heatmaply v1.4.2 in R/RStudio v4.2.2 was used to plot the resulting AAI matrix (48, 109).

### *In situ* relative abundance and growth rates

Each of the 36 metagenomes and 58 metatranscriptomes from the two bays were mapped to 18 dereplicated MAGs using Bowtie2 v2.4.5 with default parameters and converted into sorted bam files with SAMtools v1.15.1 (110, 111). Read mapping results were summarized using featureCounts v2.0.1 (38). The number of genome equivalents per metagenome was estimated using MicrobeCensus and abundance via RPKG values was calculated as previously described, based on the featureCounts table and the total length of all ORFs in each representative MAG (38–40). A bar graph was made using the ggplot2 library of R/RStudio v4.2.2 (48, 112). In addition, singleM was used to see how much of a metagenome the genomes or assembly represent (36). “corrplot” and “Hmisc” through R were used to generate a plot, showing the effects of environmental factors on average *Rhodobacterales* abundance and estimated growth rates (48, 113–115).

As a proxy to understand the replication of representative *Rhodobacterales* MAGs in sampling sites, PTR, the ratio of sequence coverage near the origin and that near the terminus of replication were obtained using the program Compute PTR (CoPTR) (47, 116) and the heatmaply package in R/RStudio was used to prepare a heatmap from the resulting data (48, 109). The normality of the CoPTR data set was checked using the Shapiro test (117). To see the effects of size fraction and environmental parameters such as season and salinity on CoPTR values of each MAG, a non-parametric Wilcoxon rank test in R was used to determine significantly different CoPTR values between genomospecies in different environments (118). A pairwise Wilcoxon test comparison was done on categories with more than two variables (e.g., seasons—spring, summer, and fall). Spearman correlations of *in situ* growth rates or abundances with environmental parameters were calculated in RStudio using linkET of the vegan package. To measure the maximal growth rates of representative genomospecies, the gRodon package in R was used to calculate codon usage bias (119). This package required Prokka v1.13 to generate gff and ffn files for each representative MAG and calculated maximal growth rates from the codon usage bias for highly expressed genes (119, 120).

### Pangenome analyses

To compare the functional potential of *Rhodobacterales* from this estuarine study with known genomes, 46 *Rhodobacterales* MAGs from this study and 57 closely related reference genomes were analyzed with DRAM (42). Also, these MAGs and genomes identified in the phylogenomic tree described above were placed into 11 pangenome groups in anvi’o v7.1 (33). The groups were named based on the genera of MAGs and genomes defined by GTDB-Tk (31, 33). Contig databases generated using anvi’o v7.1 from members of these groups were annotated internally using a gene clustering approach with HMM hits from Clusters of Orthologous Groups (COG), Kyoto Encyclopedia of Genes and Genomes (KEGG), KOfam (a customized KEGG Orthologs (KOs) database), Protein families (Pfam) databases, and externally with Prokka v1.13 (33, 120–123). A summary table was generated from anvi’o containing all gene clusters and associated annotations (33). A percentage of MAGs/genomes within a defined genera harboring a gene of interest was calculated. The matrices of gene abundance percentages in each MAG or genome as a percentage of genera were visualized as heatmaps/bubble plots in RStudio using ggplot2 packages (48, 109, 112). MAGs/genomes were designated as “estuarine,” or “marine” based on the reports about where they were found. This information was added to the pangenome as “layers” using the “anvi-import-misc-data” program in anvi’o v7.1. To see the enrichment of COG functions in either marine or estuarine MAGs/genomes, the program “anvi-compute-functional-enrichment-in-pan” in anvi’o v7.1 was used (33). Significantly enriched functions in one group (adjusted q-value < 0.05) were visualized using the ggplot2 package of RStudio (48, 112, 124). Carbohydrate-active enzymes (CAZymes) from representative MAGs were annotated using run_dbcan v4 (44–46). This program found CAZymes in MAGs by comparing their ORFs to the dbcan_seq database which provided CAZyme sequences and annotations (44). CAZymes were categorized into CAZyme families and their abundances in each of the representative MAGs were visualized in RStudio with ggplot2 (48, 112). Moreover, the dbcan-derived diamond output was manually matched with the Prodigal output to get common gene caller IDs (44–46, 125). These IDs were further used to compare gene expressions in different conditions using DESeq2 (50).

### Gene expression

Metatranscriptome reads were mapped against representative MAGs with Bowtie2 v2.4.5 as described above (110). Count tables from featureCounts of subread package v.2.0.1 were condensed based on gene clusters identified in anvi’o v7.1 per metatranscriptome (33, 38). Metatranscriptomes were used for analysis in DESeq2 if they had at least ~10,000 reads mapped to genes within a representative MAG (50). From these count tables, principal component analysis (PCA) plots were generated to see how the sample clustered regarding gene expression. For each representative MAG, differentially expressed genes were identified using subsets of feature count tables created based on temporal or spatial differences of samples. The 50 most highly expressed genes from four MAGs, that is, HI_DESum22DL08_bin_21, LF_DESum29NL08_bin_29, ME_DESum29NL08_bin_12, and Pl_DESpr20G08_bin_28 (where the first two letters indicated their respective genus names) belonging to HIMB11, LFER01, MED-G52, and *Planktomarina*, respectively, were tabulated and used for visualization. In the figures, manually edited short names/symbols of the genes from the pangenome summary were used. To determine differentially expressed genes, adjusted *P*-values were used from DESeq2 analyses (50). Visualization was done through heatmaps using DESeq2 and pheatmap packages in R (48, 50, 120).

## Data Availability

The metagenomes, metatranscriptomes, and MAGs for this study are available on NCBI under the umbrella project PRJNA432171.

## AFTER EPUB

*[This article was published on 8 January 2025 with an error in Acknowledgments. The error was corrected in the current version, posted on 7 February 2025.]*

## SUPPLEMENTAL MATERIAL

Data Set S1 aem.02357-24-s0001.xlsx Rhodobacterales MAG information with their PTR.

Data Set S2 aem.02357-24-s0002.xlsx Gene annotation of Rhodobacterales.

Data Set S3 aem.02357-24-s0003.xlsx Rhodobacterales gene expression.

Fig. S1 aem.02357-24-s0004.tiff Sampling points.

Fig. S2 aem.02357-24-s0005.tiff Representation of Rhodobacterales in samples.

Fig. S3 aem.02357-24-s0006.tiff Rhodobacterales relative abundance and environmental factors.

Fig. S4 aem.02357-24-s0007.tiff Transporter genes in Rhodobacterales.

Fig. S5 aem.02357-24-s0008.tiff CAZymes in Rhodobacterales.

Fig. S6 aem.02357-24-s0009.tiff Highly expressed genes of Planktomarina and HIMB11 genomospecies.

Supplemental material aem.02357-24-s0010.docx Table S1 and legends for Fig. S1 to S6.

ASM does not own the copyrights to Supplemental Material that may be linked to, or accessed through, an article. The authors have granted ASM a non-exclusive, world-wide license to publish the Supplemental Material files. Please contact the corresponding author directly for reuse.
