# Supplementary figures and images for: Genome-resolved adaptation strategies of Rhodobacterales to changing conditions in the Chesapeake and Delaware Bays
Source: Appl Environ Microbiol. 2025 Jan 8;91(2):e02357-24. doi: 10.1128/aem.02357-24 (PMC11837527; doi:10.1128/aem.02357-24)

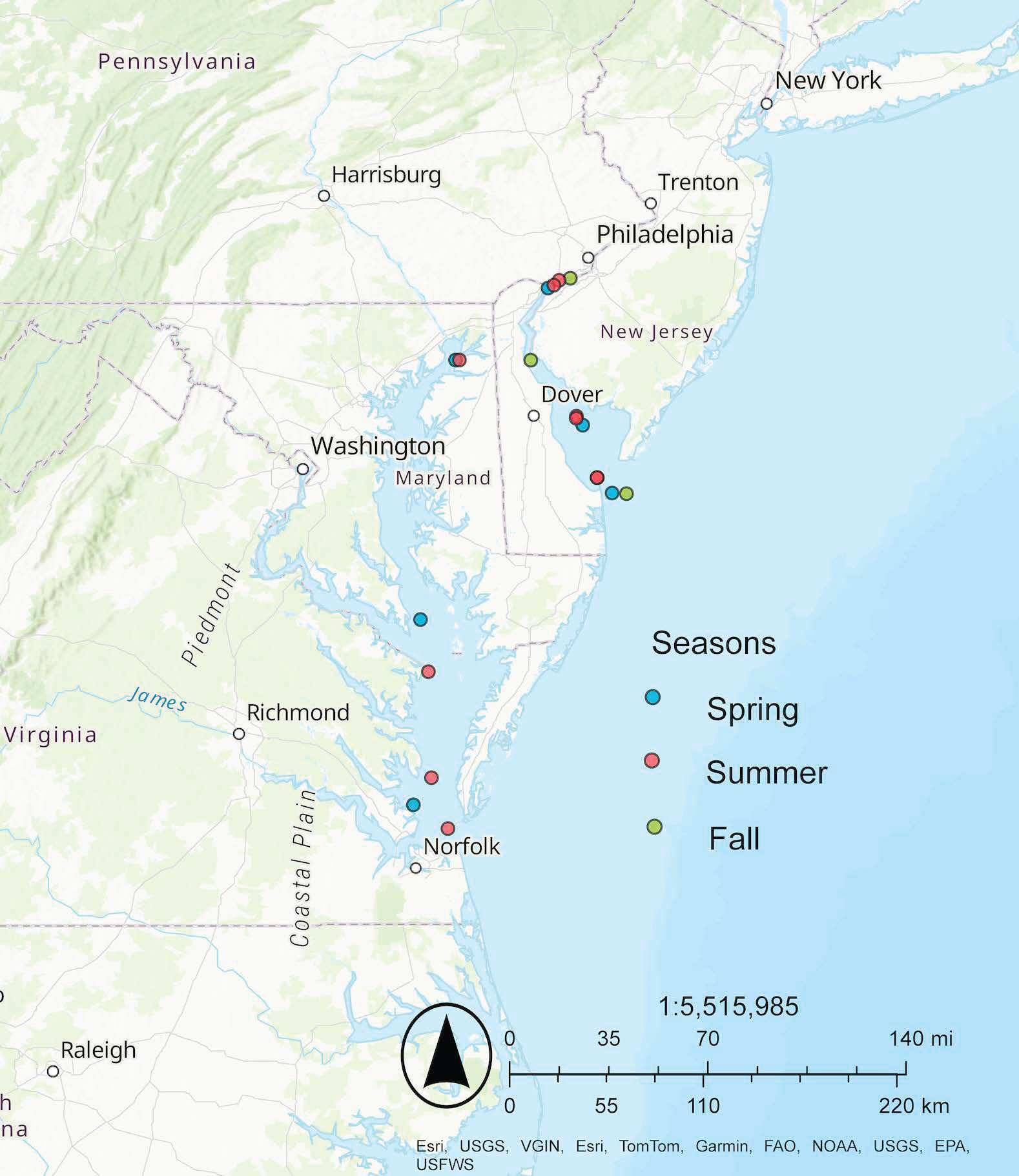

Supplement: Fig. S1 — Sampling points. [file aem.02357-24-s0004.tiff]

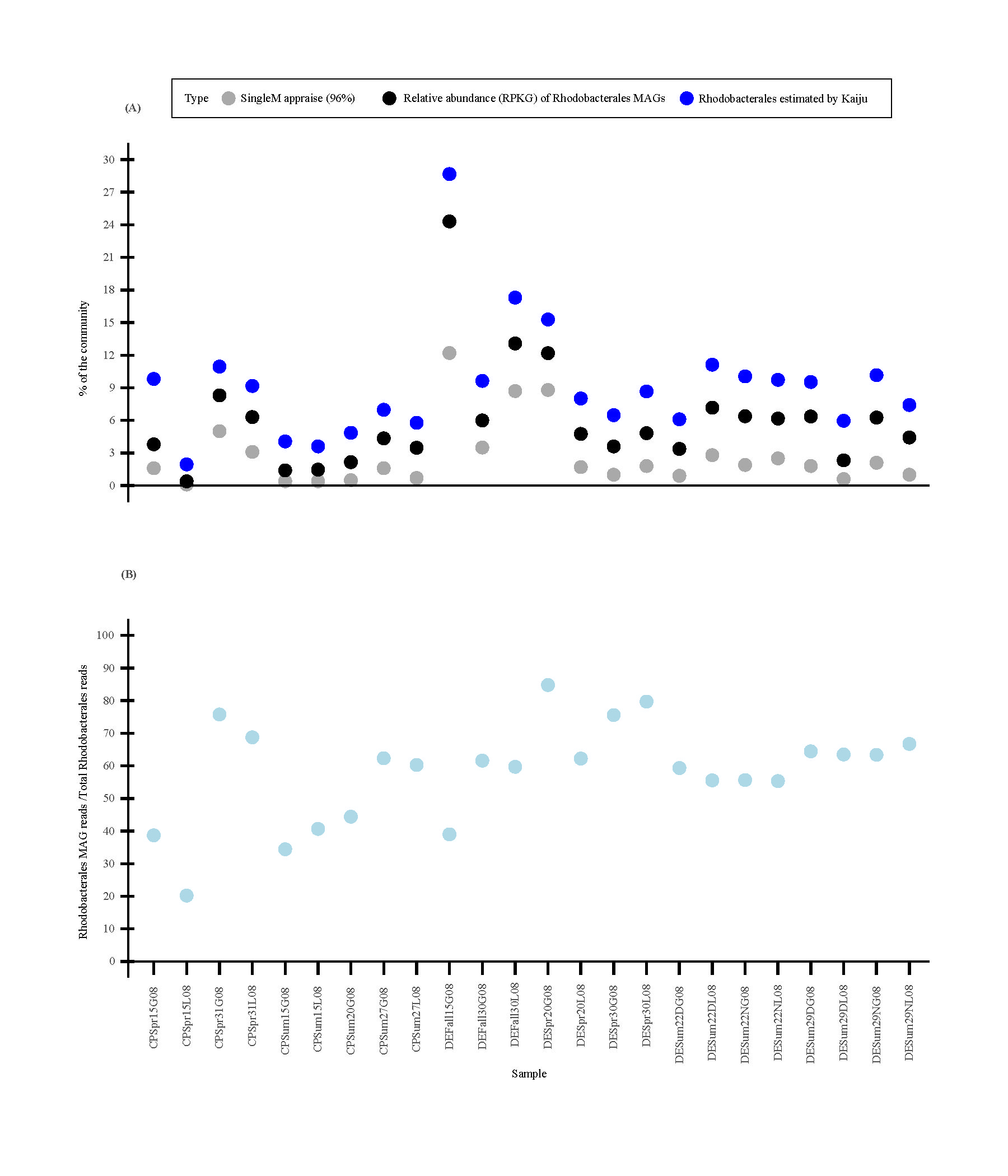

Supplement: Fig. S2 — Representation of Rhodobacterales in samples. [file aem.02357-24-s0005.tiff]

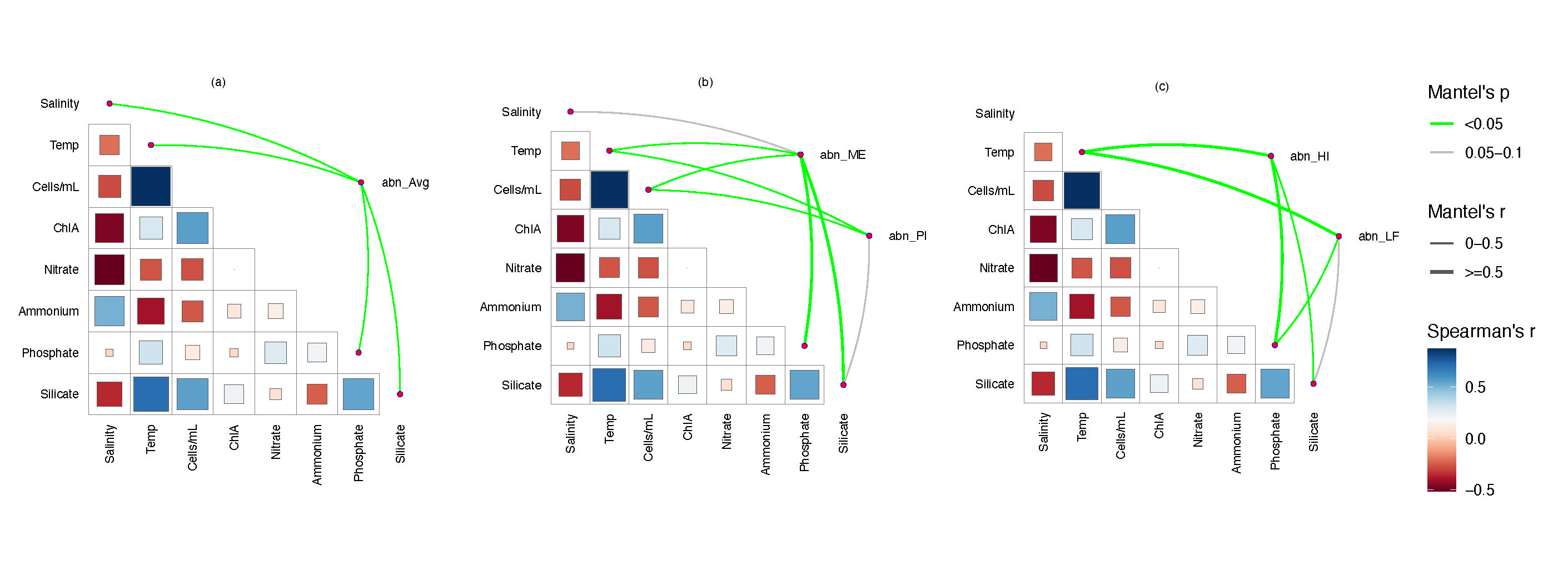

Supplement: Fig. S3 — Rhodobacterales relative abundance and environmental factors. [file aem.02357-24-s0006.tiff]

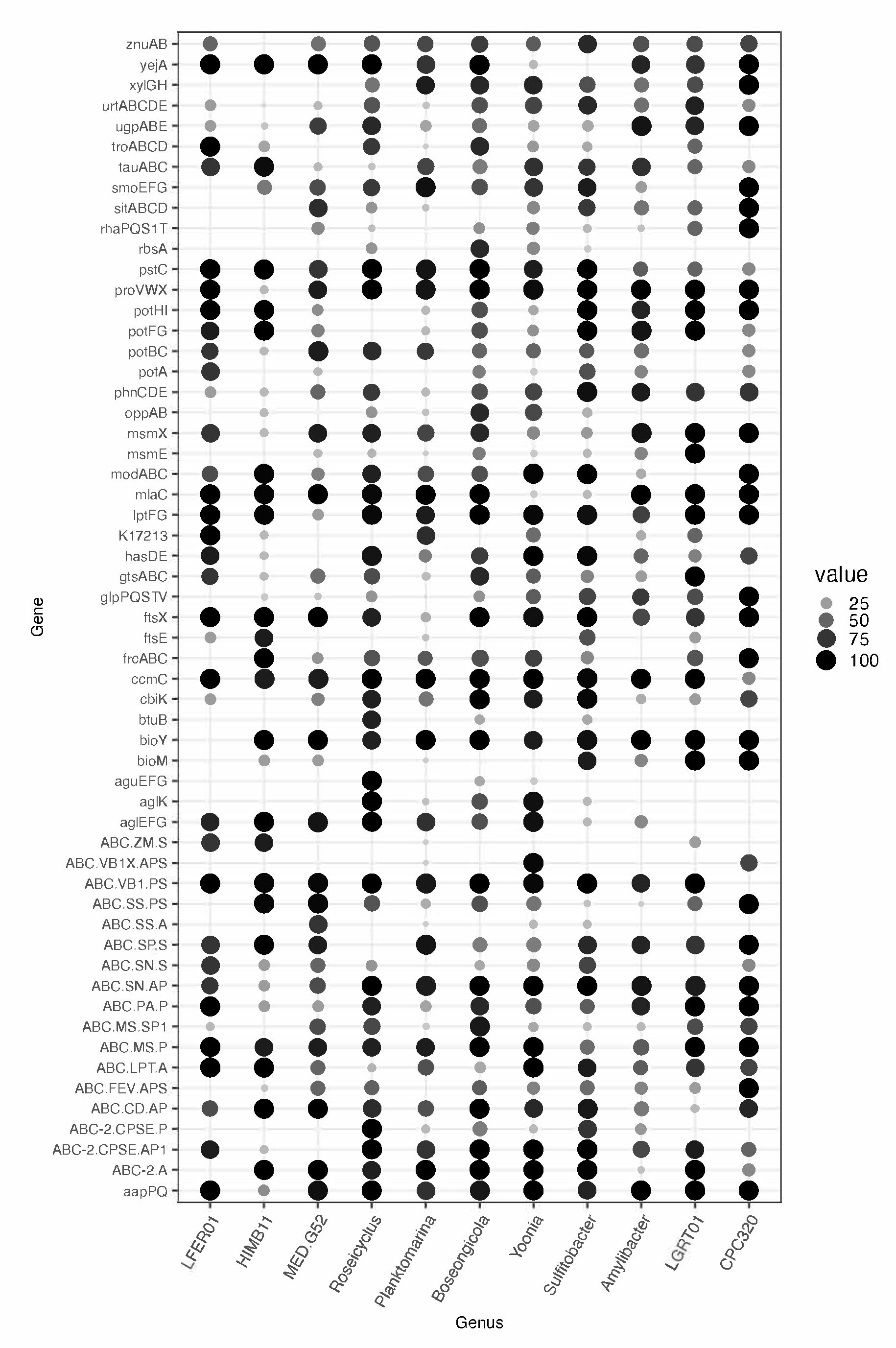

Supplement: Fig. S4 — Transporter genes in Rhodobacterales. [file aem.02357-24-s0007.tiff]

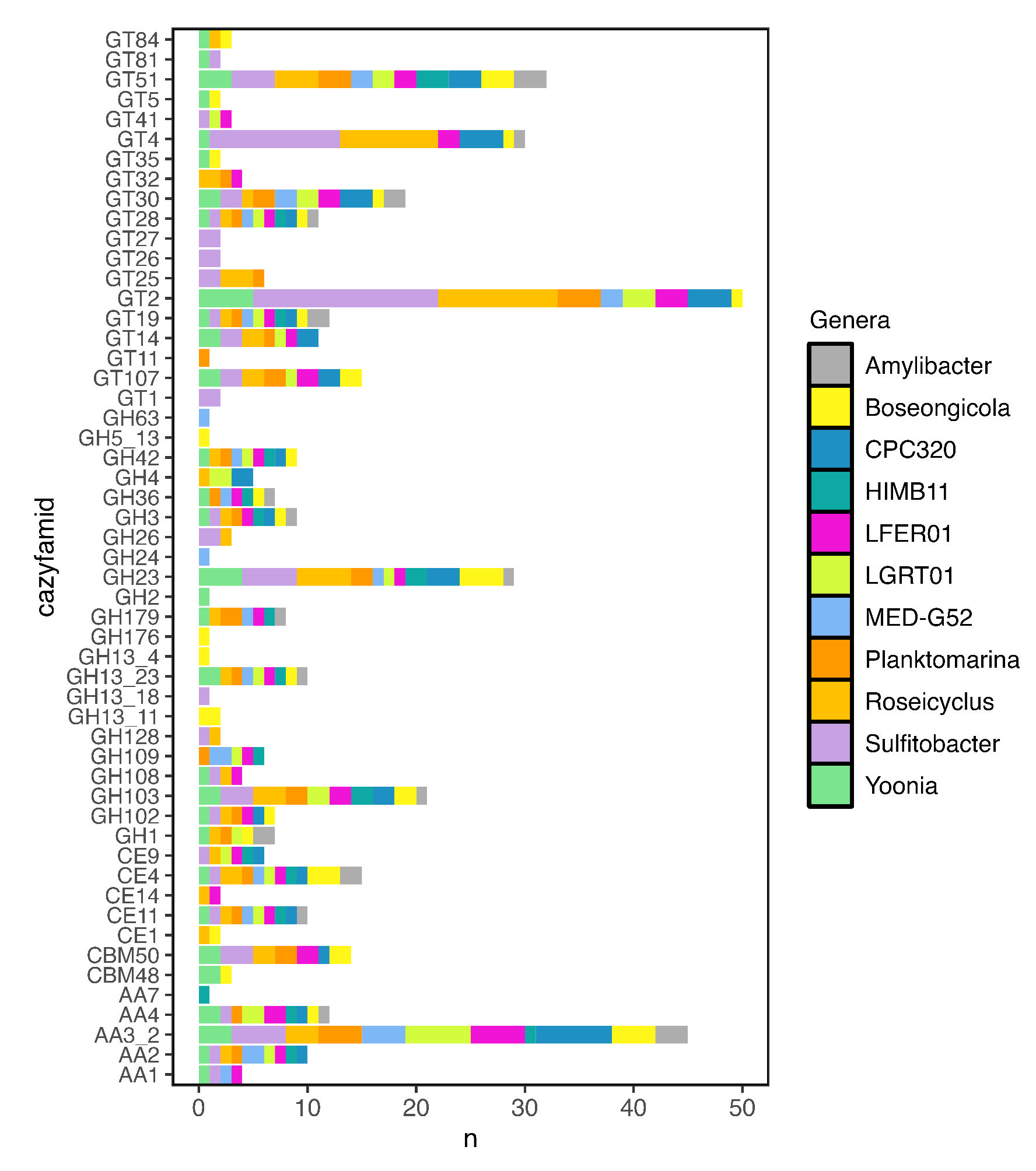

Supplement: Fig. S5 — CAZymes in Rhodobacterales. [file aem.02357-24-s0008.tiff]

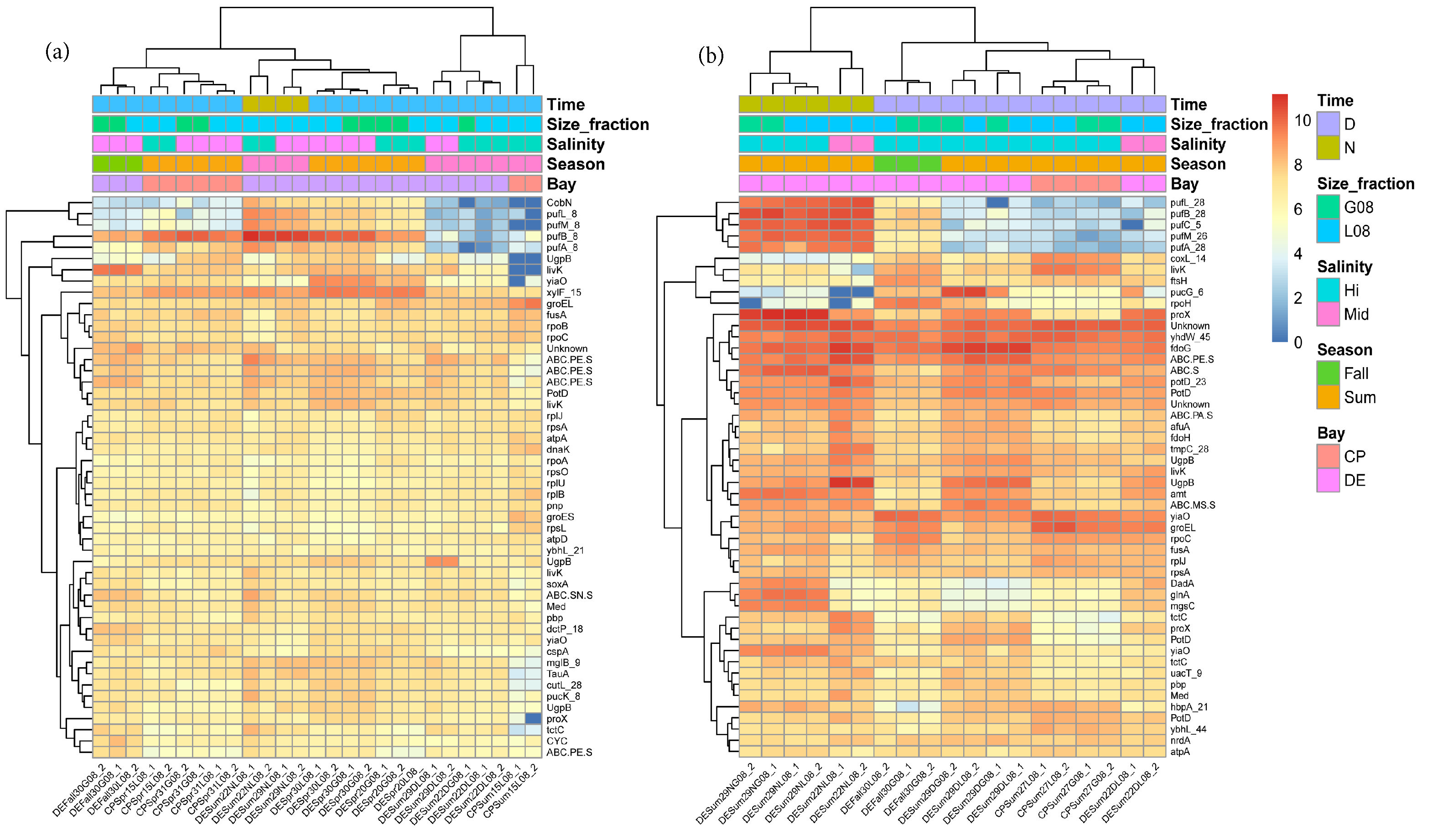

Supplement: Fig. S6 — Highly expressed genes of Planktomarina and HIMB11 genomospecies. [file aem.02357-24-s0009.tiff]
