# Supplementary material for: Genome-resolved adaptation strategies of Rhodobacterales to changing conditions in the Chesapeake and Delaware Bays
Source: Appl Environ Microbiol. 2025 Jan 8;91(2):e02357-24. doi: 10.1128/aem.02357-24 (PMC11837527; doi:10.1128/aem.02357-24)
Supplement: Supplemental material — Table S1 and legends for Fig. S1 to S6. [file aem.02357-24-s0010.docx]

**Supplemental material,**

**Title: Genome-resolved adaptation strategies of *Rhodobacterales* to changing conditions in the Chesapeake and Delaware Bays**

Running title: *Rhodobacterales* in the Chesapeake and Delaware Bays

Authors: Mir Alvee Ahmed, Barbara J. Campbell^#^

^#^Corresponding author: Barbara J. Campbell, email: [bcampb7@clemson.edu](mailto:bcampb7@clemson.edu)

**Table S1: Percentages* of differentially expressed genes of *Rhodobacterales* genomospecies in various conditions of the Chesapeake and Delaware Bays**

**Supplemental Figure 1. Sampling sites from the Chesapeake and Delaware Bays for this study.** Surface water samples were collected in different seasons from different salinity points of the Chesapeake and Delaware Bays. Salinity increased from freshwater to marine in sampling points starting from the rivers, then estuaries and the open ocean. Fall samples were only collected from the Delaware Bay.

**Supplemental Figure 2. Representation of *Rhodobacterales* in the indicated metagenomes. (A)** Relative abundance of *Rhodobacterales* MAGs defined by mapped reads compared to all classified bacterial reads; classified *Rhodobacterales* reads to all classified bacterial reads and by singleM appraise were compared to estimate the abundance of *Rhodobacterales* in our data (33, 34). **(B)** The proportion of recovered *Rhodobacterales* reads present in the MAGs compared to all classified *Rhodobacterales* reads in the indicated metagenome.

**Supplemental Figure 3. Correlation plot between environmental factors and *Rhodobacterales* MAG abundance. (a)** Average abundance (abn_Avg) and environmental parameters for each sample were analyzed for Mantel’s correlation coefficients and *p*-values using the vegan package in RStudio (41, 106). Separate correlations of environmental factors and relative abundances of **(b)** *Planktomarina* and MED-G52, **(c)** HIMB11 and LFER01 were also analyzed.

**Supplemental Figure 4. Transporters encoding genes within each of the eleven *Rhodobacterales* genera.** Percentages of members in each genus carrying the gene for transporters on the y-axis were calculated from the pangenome summary table annotated with DRAM (121) (Supplemental Data). Genes common to all genera are not shown.

**Supplemental Figure 5.** **Carbohydrate-Active Enzymes (CAZyme) genes in representative MAGs.** CAZymes from eleven genera were analyzed with run_dbcan v4 (38), were defined into families shown on the y-axis. Abbreviations: AA= auxiliary activities; CBM = carbohydrate-binding modules; CE = carbohydrate esterases; GH = glycoside hydrolases; GT = glycosyl transferases. # after the short CAZyme family IDs indicate subfamilies.

**Supplemental Figure 6. Heat maps showing the top 50 highly expressed genes in varying conditions. (a)** ME_DESum29NL08_bin_12 and **(b)** ME_DESum29NL08_bin_12. DESeq2 was used to find top 50 highly expressed genes of these genomospecies (129). Abbreviations: CP = Chesapeake Bay; DE = Delaware Bay; Spr = Spring; Sum = Summer; # = salinity in PSU; G08 = >0.8 µm and L08 = <0.8 µm size-fractions; # = RNA1 or RNA2; D = Day; N = Night. Gene symbols are defined in the Supplemental Data.

**Table S1: Percentages* of differentially expressed genes of *Rhodobacterales* genomospecies in various conditions of the Chesapeake and Delaware Bays**

| **Conditions** | **Pl_28** | **HI_21** | **ME_12** | **LF_29** | **Median** |
| --- | --- | --- | --- | --- | --- |
| DESumHiMidL08_NvsD | 2.4 | 6.6 | 6.6 | 10 | 6.6 |
| DESumMidL08_NvsD | 2.6 | 3.6 | 6.1 | 10.4 | 4.9 |
| DESumHiL08_NvsD | 2.4 | 4.9 | 7.1 | 5.3 | 5.1 |
| DESumHiG08NvsD |  | 7.6 | 5.5 |  | 6.5 |
| DEHiG08_FallvsSpr | 9.5 |  |  |  | 9.5 |
| DEHiG08SumvsFall |  | 4.1 |  |  | 4.1 |
| CPSprHiL08vsG08 | 0.7 |  |  |  | 0.7 |
| CPSprL08MidvsHi | 5.8 |  |  |  | 5.8 |
| CPSumMidL08vsG08 |  | 7.4 |  |  | 7.4 |
| DESumMidL08vsG08 |  | 0 |  |  | 0 |
| SumHiG08DEvsCP | 8.3 | 8.4 | 9.1 |  | 8.4 |
| SumMidL08DEvsCP | 3.3 | 5.5 |  | 13.9 | 5.5 |
| DESprHiL08vsG08 | 0 |  |  |  | 0 |
| SprHiG08DEvsCP | 7.8 |  |  |  | 7.8 |
| SumMidG08DEvsCP |  | 4.4 |  |  | 4.4 |
| CPSumHiL08vsG08 |  |  | 6.7 |  | 6.7 |
| **Median** | 3 | 5.2 | 6.6 | 10.2 |  |

* A percentage of differentially expressed genes among total annotated genes was calculated for four genomospecies in various environmental conditions. For example, DESumHiMidL08_NvsD means the differential expression of genes in day vs. night in the Delaware Bay (DE), summer (sum), high and medium salinity (HiMid), small size-fraction (L08) samples.

Abbreviations: CP = Chesapeake Bay; DE = Delaware Bay; Spr = Spring; Sum = Summer; Hi and Mid = High and medium salinity; D = day; N = night; G08 = >0.8 µm and L08 = <0.8 µm size-fractions; Pl_28 = Pl_DESpr20G08_bin_28; HI_DESum22DL08_bin_21; ME_12 = ME_DESum29NL08_bin_12, and LF_29 = LF_DESum29NL08_bin_29).
